# Minimising pacemaker exposure in thoracic radiotherapy: a comparative study of IMRT, VMAT, and helical tomotherapy

**DOI:** 10.1186/s43046-026-00369-4

**Published:** 2026-06-13

**Authors:** Sandeep Singh, Supratik Sen, Abhay Kumar Singh, Dipesh, Manindra Bhushan, Anuj Vijay, Benoy Kumar Singh, Raj Pal Singh, Munish Gairola

**Affiliations:** 1 Department of Radiation Oncology, Amrita School of Medicine, Amrita Vishwa Vidyapeetham, Faridabad, India; 2 Department of Physics, GLA University, Mathura, India; 3 Department of Radiation Oncology, Bhubaneswar Borooah Cancer Institute, Guwahati, India; 4 Department of Radiation Oncology, Dharamshila Narayana Super speciality Hospital, New Delhi, India; 5 Department of Radiation Oncology, Andromeda Cancer Hospital, Kundli, Haryana India; 6 Department of Radiation Oncology, Division of Medical Physics, Rajiv Gandhi Cancer Institute and Research Centre, New Delhi, India; 7 Department of Radiation Oncology, Rajiv Gandhi Cancer Institute and Research Centre, New Delhi, India

**Keywords:** Pacemaker, CIED, Dose to generator, OAR sparing, Field-width modulation

## Abstract

**Purpose:**

Patients with cardiac implantable electronic devices (CIEDs) increasingly require thoracic radiotherapy, but comparative evidence guiding technique selection remains limited. This study evaluated CIED dose, target coverage, organ-at-risk (OAR) sparing, and delivery efficiency across IMRT, VMAT, and helical tomotherapy in matched thoracic cases.

**Methods:**

Eighty patients with left-sided subclavicular pacemakers were planned using five techniques: IMRT, VMAT, and tomotherapy with 1 cm, 2.5 cm, and 5 cm field widths. All plans used 6 MV FFF photons with harmonised dose constraints. CIED endpoints included Dmax, D0.03 cc, and Dmean. Secondary endpoints included PTV coverage, homogeneity, conformity, lung and heart dose metrics, monitor units, and beam-on time. Statistical comparisons were performed using repeated-measures ANOVA with Holm-Bonferroni correction.Out-of-field dose was validated using OSLDs in a RANDO phantom.

**Results:**

Target coverage was comparable across techniques (PTV D95 ≈ 98.5–99.2%). CIED dose was significantly reduced with tomotherapy, with mean Dmax decreasing from 1.86 ± 0.43 Gy (IMRT) and 1.68 ± 0.38 Gy (VMAT)to 1.10 ± 0.25 Gy for the 1 cm field width (*p* < 0.01). Mean heart dose decreased from 8.2 Gy to 3.8 Gy, and mean lung dose from 9.4 Gy to 7.6 Gy. VMAT required fewer monitor units (500 vs. 910 for IMRT) and shorter delivery (1–1.5 min), whereas tomotherapy ranged from 140 to 500 s.

**Conclusion:**

Helical tomotherapy improves CIED sparing and selected OAR metrics while maintaining target coverage. However, VMAT offers superior delivery efficiency. Technique selection should therefore be individualised, balancing dosimetric benefit against intrafraction motion risk, target geometry, and patient-specific clinical priorities.

## Introduction

The increasing coexistence of thoracic malignancies and cardiac implantable electronic devices (CIEDs), including pacemakers and implantable cardioverter-defibrillators, has created a growing clinical challenge in modern radiotherapy practice. Because these devices remain susceptible to ionising radiation, careful consideration of treatment technique and dose exposure has become increasingly importan [1]. Radiation exposure to CIEDs can result in device reset, oversensing, pacing inhibition, or, in rare cases, permanent malfunction. These effects may arise from direct irradiation of the generator or leads, electromagnetic interference generated during beam delivery, or neutron-related interactions at higher beam energies. In most thoracic treatments, the dose received by the pacemaker is dominated not by the primary treatment beam, but by out-of-field scatter and treatment-head leakage, which can accumulate progressively over multiple fractions [2].

Current professional recommendations generally emphasize minimizing CIED dose as much as reasonably achievable, implementing enhanced monitoring when higher exposure is expected, and avoiding unnecessary use of high-energy photon beams. Although several reports identify approximate cautionary thresholds near 2 Gy and recommend closer surveillance beyond 5 Gy, there remains no universally accepted guideline defining a single optimal radiotherapy planning technique for patients with implanted cardiac devices [3]. Consequently, the selection of treatment modality remains largely institution-dependent, often dictated by equipment availability, clinical experience, and the spatial relationship among the target volume, adjacent organs at risk (OARs), and the implanted device.

Modern platforms differ in ways that plausibly matter for CIED dose. On a C-arm linac, intensity-modulated radiotherapy (IMRT) uses a limited set of fixed gantry angles. The geometry allows explicit beam avoidance of the device, but can drive up monitor units [4]. Volumetric modulated arc therapy (VMAT) achieves conformity and efficiency through continuous rotation with dynamic MLC and dose-rate changes. Unless arcs are used with an avoidance sector, the low-dose bath tends to broaden [5]. Tomotherapy offers two distinct delivery methods: Tomo Direct (utilising multiple fixed fan-beam fields) and helical delivery (employing a continuous spiral motion). Helical plans are renowned for coverage and gradient control, yet their rotational nature can increase out-of-field spread compared with carefully selected fixed-field strategies [6, 7].

Despite numerous case reports and precaution checklists, there is little head-to-head evidence that puts the device itself at the centre of comparison across machines and techniques. Specifically, systematic data contrasting IMRT, VMAT, and helical tomotherapy for pacemaker dose in thoracic sites are scarce. This gap leaves planners to extrapolate from general principles about scatter and rotation rather than from direct measurements or uniform plan evaluations.

Contemporary management of CIED during radiotherapy is anchored by AAPM task group recommendations, which have evolved from early conservative advice to a structured, risk-based framework. TG-34 first emphasised minimising dose to the pulse generator and maintaining vigilance when any nontrivial exposure was anticipated [8]. TG-203 updates this approach by defining risk categories according to cumulative generator dose, low risk for exposures < 2 Gy, intermediate for 2–5 Gy, and high risk for > 5 Gy, and by highlighting that neutron-producing energies (e.g., ≥ 10 MV photons) can precipitate device malfunctions even when the recorded dose to the generator is modest [3]. TG-203 also differentiates risk by device class and dependency. ICDs are generally more susceptible than pacemakers, and patients who depend on pacemakers warrant tighter safeguards. Because leads lack active electronics, the report does not prescribe separate dose limits for the wires. Instead, it concentrates on sparing the generator, avoiding unnecessarily high beam energies, and scaling surveillance measures to the expected exposure. In practical terms, the guidance translates to keeping the generator dose as low as reasonably achievable, favouring ≤ 6 MV photons when clinically feasible, estimating and documenting cumulative out-of-field dose (which often arises from head leakage and scatter rather than the primary field), and intensifying monitoring as the projected dose moves beyond 2 Gy. This includes cardiology involvement and real-time ECG oversight when doses may exceed 5 Gy or when high-energy beams are unavoidable.

This study aims to bridge that gap. Using matched thoracic datasets and harmonised prescriptions and OAR limits, we will compare device-focused endpoints with standard plan quality indices, and treatment efficiency. We had validated treatment-planning estimates with measurements around a pacemaker surrogate in an anthropomorphic phantom. We hypothesised that delivery geometry, beam modulation strategy, and tomotherapy field width would significantly influence CIED dose, even when target coverage and OAR constraints were harmonised across techniques.

## Materials and methods

### Patient cohort

We selected 80 lung and oesophageal cancer cases that reflected typical thoracic presentations, with the CIED positioned in a realistic subclavicular pocket (left). Simulation CTs were acquired supine with arms elevated on a customised thoracic board, using 3-mm slices and a scan extent from the cricoid to L2. When available, diagnostic CT and PET/CT were rigidly co-registered to guide target and OAR delineation. To avoid neutron production and standardise comparisons, all plans were generated using 6 MV flattening-filter-free (FFF) photon beams. Because tomotherapy inherently delivers treatment with a 6 MV FFF beam, the same beam quality was selected on the C-arm LINAC to ensure a methodologically fair and direct dosimetric comparison.

### Contouring and structures

Clinical target volumes were delineated on contrast-enhanced CT to include the primary lesion and involved lymph nodes, following ASTRO ACROP guidelines for lung and oesophageal cancer [9]. A planning target volume (PTV) margin of 5 mm was applied according to the technique. For every case, the CIED was fully visualised on the planning CT and contoured. Institutional constraints limited the cumulative CIED dose to ≤ 2 Gy, and all devices were kept outside primary beam fields. Treatment was prescribed to the PTV at 41.4 Gy in 23 fractions (1.8 Gy/fx). Plans were normalised to ensure that ≥ 95% of the PTV received ≥ 95% of the prescribed dose. The mean PTV volume across the cohort was 286 ± 35.8 cm³, reflecting variability in tumour size and extent.

### Technique-specific planning parameters

**Fig. 1:**
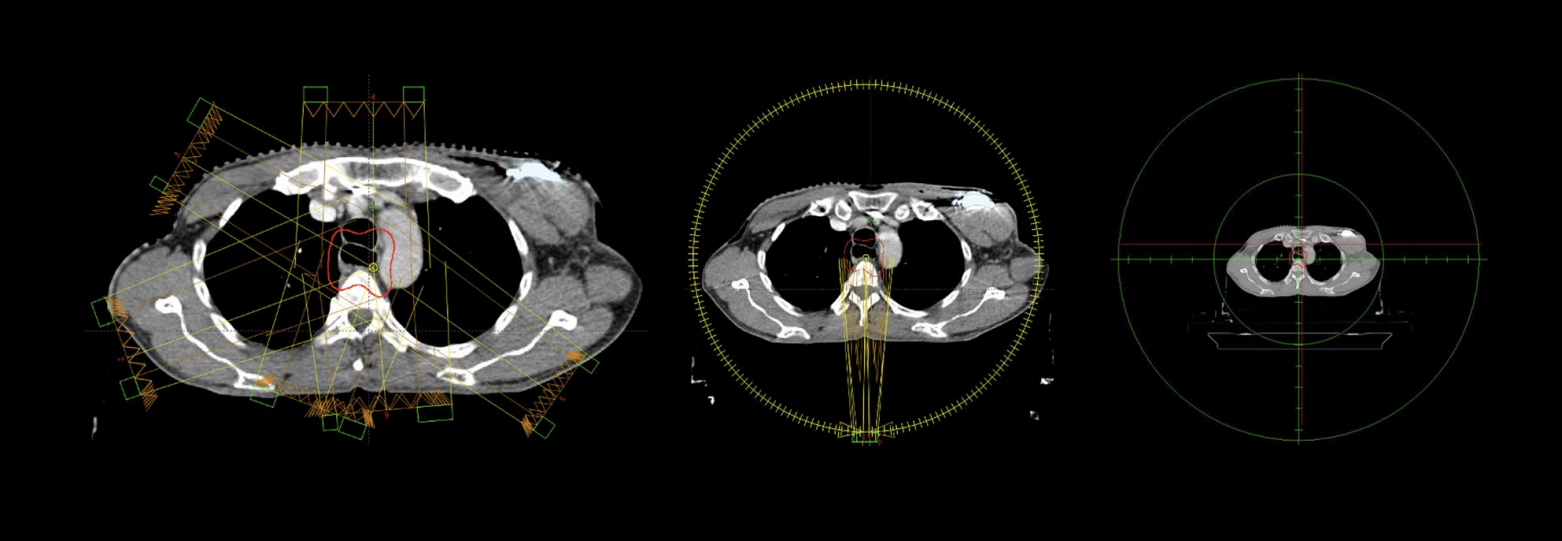
Shows axial CT images comparing beam geometry and dose delivery characteristics across three planning techniques for a thoracic case. (Left) Static-field IMRT showing multiple fixed gantry angles converging on the target volume. (Middle) VMAT plan depicting dynamic MLC modulation with continuous gantry rotation around the PTV. (Right) Helical plan illustrating a continuous spiral delivery pattern achieved through simultaneous couch translation and gantry rotation. These images demonstrate the differing beam arrangements and modulation strategies that influence dose conformity and treatment efficiency

IMRT and VMAT plans were generated on a Varian TrueBeam STx (Varian Medical Systems, Palo Alto, USA) linear accelerator equipped with HD120 MLCs using 6 MV FFF photons. IMRT plans used 5–7 coplanar fields, with gantry angles selected based on PTV geometry and arranged to avoid direct beam entry or exit through the CIED where feasible. Collimator angles ranged from 10° to 30°, and optimisation prioritised target coverage followed by OAR sparing and explicit minimisation of CIED dose (Fig. 1 (Left)). VMAT plans were generated using two to three coplanar arcs (full or partial), with collimator angles of 15°–35°. Avoidance sectors were incorporated around the CIED region to reduce entrance fluence while maintaining adequate target coverage and OAR sparing. Optimisation followed the same priority hierarchy, with particular emphasis on minimising CIED dose (Fig. 1(Middle)). Helical tomotherapy plans were generated on a Radixact system using Accuray Precision (version 3.3.1.3 (2)) planning system (Fig. 1 (Right)). For each patient, three plans were created with field widths of 1 cm, 2.5 cm, and 5 cm to evaluate the effect of field width on dosimetric outcomes. Pitch, defined as the ratio of couch travel per gantry rotation to field width, was fixed at 0.287 to minimise thread effects, and the modulation factor was set to 2.5 to ensure adequate intensity modulation with acceptable delivery time. Dynamic jaw mode was used for 2.5 cm and 5 cm field widths, while the 1 cm plan was generated using fixed jaw mode. Optimisation objectives and constraints were kept identical across all plans. All plans were generated by the same experienced planning team using a standardised optimisation framework prioritising PTV coverage, OAR sparing, and CIED protection. Plans were independently reviewed by a senior physicist to ensure consistency and quality. Dose calculations for all plans were performed using clinically commissioned algorithms specific to each treatment platform. IMRT and VMAT plans on the C-arm LINAC were calculated using the Anisotropic Analytical Algorithm (AAA) within the Eclipse treatment planning system (v15.1), whereas helical plans were generated using the convolution–superposition algorithm. All algorithms were commissioned and validated in accordance with institutional protocols and relevant guidelines to ensure accurate dose calculation. As the primary objective of this study was to compare delivery techniques rather than dose calculation algorithms, each modality was evaluated using its respective standard clinical algorithm.

### Optimisation objectives and constraints

For the PTV, plans were optimised so that at least 95% of the PTV received at least 95% of the prescription dose, while limiting D2% to ≤ 107% of the prescription dose and minimising dose heterogeneity [10]. For organs at risk, limit the spinal cord maximum dose to Dmax ≤ 45 Gy; for the oesophagus, aim for Dmean ≤ 34 Gy and D0.1 cc ≤ 60 Gy; for the lungs minus PTV, keep Dmean ≤ 15 Gy with V20 ≤ 30% and V5 minimised; and for the heart, target Dmean within 10–15 Gy and D0.1 cc ≤ 60 Gy.

**Fig. 2:**
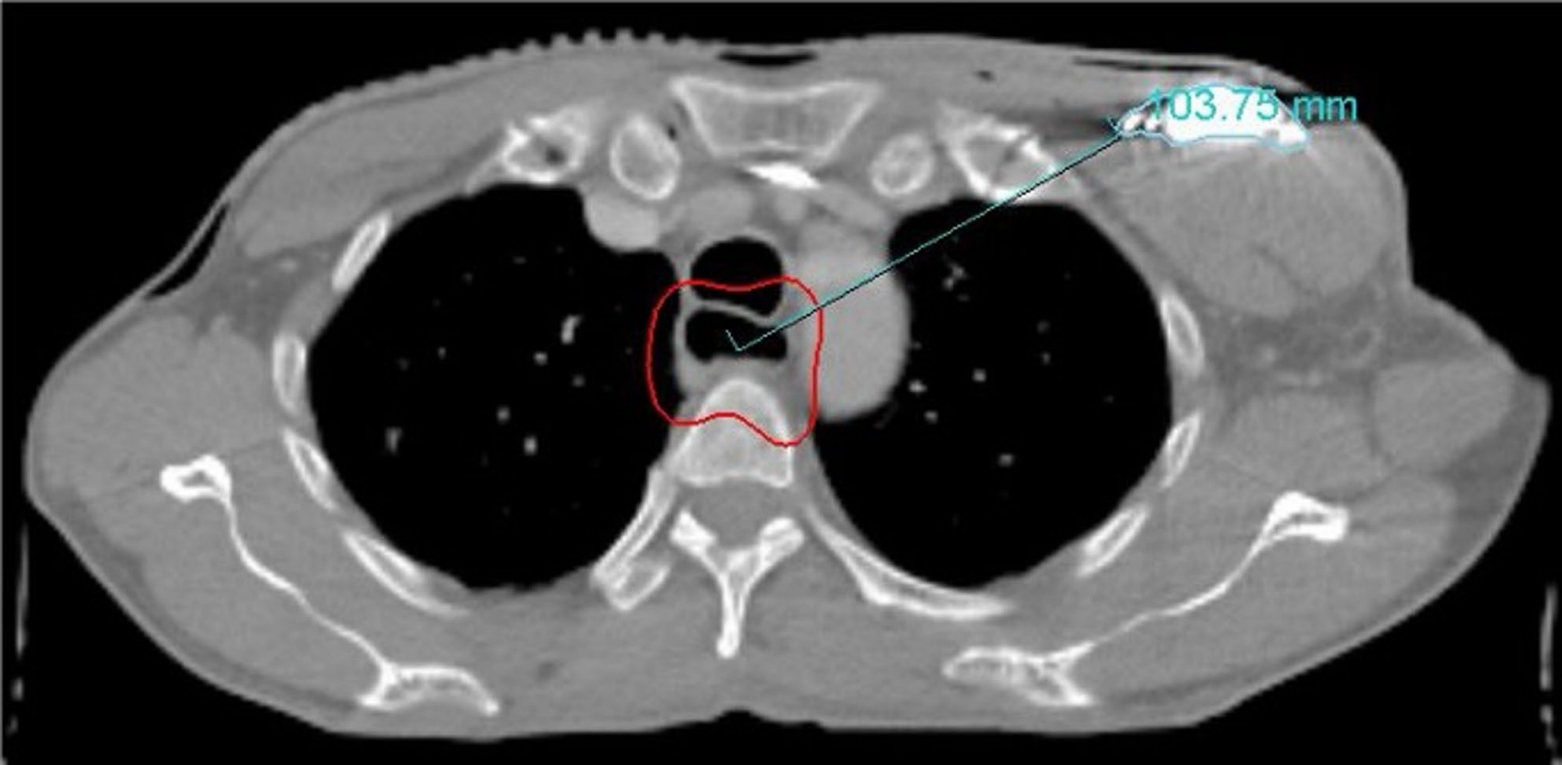
Shows an axial CT image depicting the measurement of distance from the geometric centre of the PTV (outlined in red) to the surface of the implanted pacemaker device. Primary endpoints for the CIED were Dmax (point), D0.03 cc, and Dmean. Secondary endpoints included organ-specific dose metrics, standard plan quality indices (PTV D95, conformity index, HI), and efficiency (monitor units and beam-on time). All DVHs were exported at a resolution of 0.01 Gy. Plans were considered acceptable only if PTV coverage and significant OAR limits were met

For the CIED, the primary planning objective was to minimize D0.03 cc while maintaining the cumulative generator dose below 2 Gy under conventional fractionation. To further limit local scatter and beam intensity near the generator, an optimization penalty was applied using a concentric CIED-ring structure extending from 5 mm to 15 mm beyond the device contour, thereby discouraging fluence immediately adjacent to the hardware. In addition, the shortest three-dimensional distance between the CIED centroid and the PTV surface was recorded for each case (Fig. 2) and included as a predefined variable for sensitivity analysis.

### Phantom validation of out-of-field dose

An anthropomorphic thorax phantom (RANDO) was used to simulate pacemaker conditions by placing a square lead-wire enclosure on the anterior chest surface. OSLDs were positioned inside this lead-wire box and at a depth of 1 cm within the phantom. For each delivery technique, a single representative plan was generated and delivered to the phantom five times. Dose was recorded using OSLDs, which were pre-calibrated on-axis at 6 MV and corrected for individual detector response. The measured OSLD doses at both locations were compared directly with TPS-predicted values at corresponding points [11–14].

### Statistics

Because each patient generated five paired plans, data normality was evaluated for every parameter using the Shapiro–Wilk test. Depending on the distribution, either a repeated-measures ANOVA (for normally distributed data) or the Friedman test (for non-parametric data) was applied to compare techniques. When overall significance was detected, pairwise comparisons were performed using the paired t-test (parametric) or the Wilcoxon signed-rank test (non-parametric). To control for multiple testing, adjusted p-values were calculated using the Holm–Bonferroni correction, while the Benjamini–Hochberg false discovery rate method was used for confirmatory comparisons of secondary endpoints. Effect sizes were reported as partial η² for ANOVA, Kendall’s W for Friedman, Cohen’s d_z_ for paired t-tests, and rank-biserial correlation for Wilcoxon tests. All analyses were conducted in Python (v3.10.9) using the SciPy and stats models libraries, with statistical significance set at adjusted *p* < 0.05 [15].

## Results

### PTV dose metrics

**Fig. 3:**
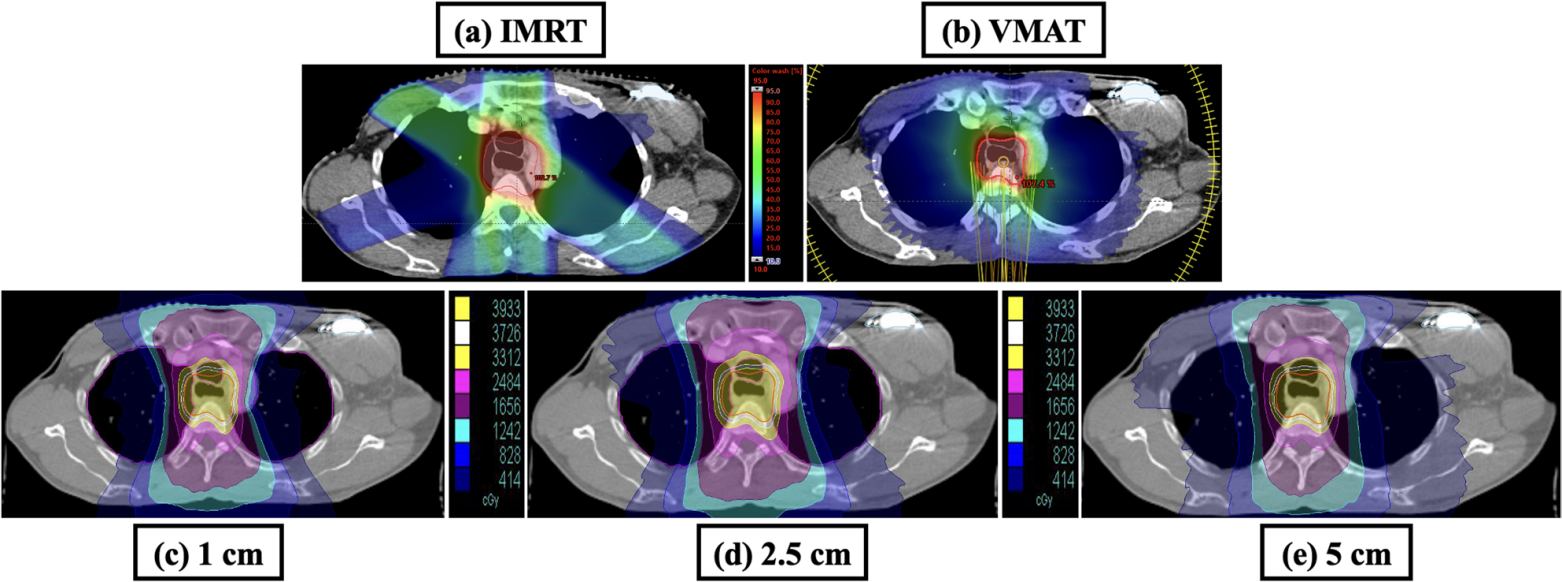
Shows axial dose distribution across different plans. (**a**) IMRT plan showing multiple fixed field arrangements. (**b**) VMAT plan demonstrating continuous arc delivery with dynamic modulation. (**c**–**e**) Helical plans with varying field widths of 1 cm, 2.5 cm, and 5 cm, respectively, illustrate the impact of jaw width on dose conformity, target coverage, and peripheral dose spillage

**Fig. 4:**
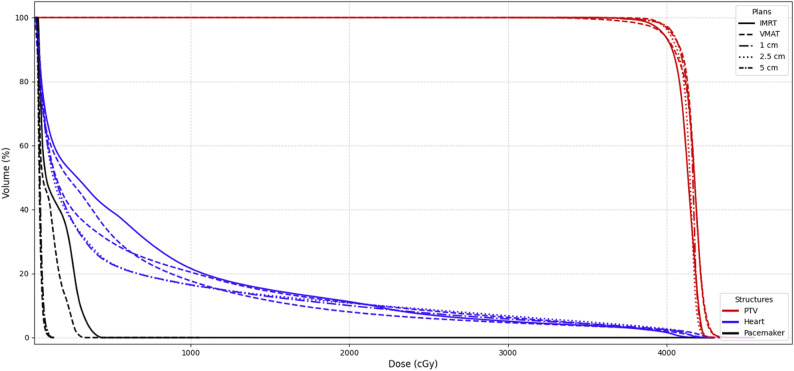
Shows DVH comparing target coverage and dose fall-off among different planning techniques. Curves represent IMRT (solid blue), VMAT (dashed yellow), and Helical plans with field widths of 1 cm (green dash-dot), 2.5 cm (purple dotted), and 5 cm (red dashed). The plot illustrates differences in dose conformity, gradient, and low-dose spillage, with narrower field widths demonstrating steeper gradients and improved dose fall-off compared to broader field configurations

Across all five planning techniques, target coverage was achieved consistently, with all plans meeting the prescribed dose criteria (Table 1; Fig. 3). Although statistical analysis demonstrated significant differences in PTV dose parameters, these variations were small and not clinically meaningful. Helical plans, particularly with smaller field widths, showed a tendency toward improved dose uniformity within the target, whereas C-arm LINAC–based techniques maintained comparable coverage with slightly higher dose heterogeneity (Fig. 4). Overall, all techniques provided acceptable and clinically equivalent target dose coverage.

**Table 1:** Shows summary of target coverage, organ-at-risk dose metrics, CIED dose parameters, and PTV–CIED spatial relationship across all treatment techniques. Values are presented as mean ± standard deviation. Dose parameters are reported in Gy, lung dose–volume metrics as percentages, and PTV–CIED distance in millimeters. PTV dose metrics include D98, D95, D50, and D2. Organ-at-risk evaluation included lung V5, V10, V20, mean lung dose, heart Dmean, heart D0.1 cc, and spinal cord Dmax. CIED dose assessment included Dmax, Dmean, and D0.03 cc. The PTV–CIED distance remained consistent across all techniques, indicating comparable anatomical geometry throughout the cohort

Parameter	IMRT	VMAT	Tomo 1 cm	Tomo 2.5 cm	Tomo 5 cm
PTV D98 (Gy)	38.79 ± 0.36	39.66 ± 0.10	40.69 ± 0.13	39.60 ± 0.04	38.50 ± 0.20
PTV D95 (Gy)	39.57 ± 0.16	40.38 ± 0.10	41.28 ± 0.04	40.35 ± 0.11	39.73 ± 0.13
PTV D50 (Gy)	41.45 ± 0.08	41.62 ± 0.11	41.75 ± 0.13	41.67 ± 0.06	41.73 ± 0.10
PTV D2 (Gy)	42.54 ± 0.19	42.83 ± 0.06	42.40 ± 0.06	42.30 ± 0.03	42.86 ± 0.06
Lung V5 (%)	57.46 ± 1.52	58.98 ± 2.56	49.46 ± 0.49	57.49 ± 0.56	60.46 ± 0.84
Lung V10 (%)	39.12 ± 1.22	40.48 ± 1.25	21.37 ± 0.68	30.99 ± 0.73	46.18 ± 1.27
Lung V20 (%)	11.03 ± 1.14	7.58 ± 0.60	7.21 ± 0.19	9.35 ± 0.20	9.20 ± 0.23
Mean lung dose (Gy)	9.37 ± 0.35	8.91 ± 0.10	7.63 ± 0.45	8.43 ± 0.05	8.50 ± 0.10
Heart Dmean (Gy)	8.17 ± 0.85	6.09 ± 0.08	3.77 ± 0.21	5.53 ± 0.05	6.46 ± 0.06
Heart D0.1 cc (Gy)	41.66 ± 0.10	42.74 ± 0.09	42.13 ± 0.06	42.22 ± 0.08	42.32 ± 0.07
Cord Dmax (Gy)	23.22 ± 1.44	17.45 ± 0.62	25.70 ± 0.56	26.19 ± 0.60	27.75 ± 0.11
CIED Dmax (Gy)	1.86 ± 0.43	1.68 ± 0.38	1.10 ± 0.25	1.25 ± 0.27	1.34 ± 0.30
CIED Dmean (Gy)	0.92 ± 0.21	0.81 ± 0.18	0.55 ± 0.12	0.61 ± 0.14	0.67 ± 0.16
CIED D0.03 cc (Gy)	1.42 ± 0.31	1.29 ± 0.27	0.88 ± 0.20	0.97 ± 0.22	1.05 ± 0.24
PTV–CIED distance (mm)	45.8 ± 4.2	45.6 ± 4.1	45.7 ± 4.3	45.9 ± 4.0	45.8 ± 4.1

### CIED dose and distance relationship

Dose to the CIED varied significantly across planning techniques, with helical consistently demonstrating reduced device dose compared to IMRT and VMAT (Table 1). Statistical analysis confirmed a significant difference among techniques, with post hoc comparisons showing a lower CIED dose across all tomotherapy configurations. Although the CIED dose is inherently influenced by its spatial relationship to the target, the PTV–CIED distance was consistent across the cohort, indicating that the observed reduction was primarily attributable to differences in delivery technique rather than anatomical variation.

### Organ-at-risk doses and normality assessment

Organ-at-risk sparing demonstrated a clear dependence on treatment technique (Table 1). Helical plans, particularly with smaller field widths, provided improved sparing of critical structures, including the heart and lungs, with reductions observed across both mean dose and low- to intermediate-dose lung volumes. VMAT showed comparable or improved performance in certain intermediate-dose lung metrics, while IMRT generally resulted in higher dose exposure to normal tissues. The spinal cord dose remained within acceptable limits across all techniques, with comparatively lower values observed with VMAT. Overall, these findings indicate that helical delivery enhances normal tissue sparing, although the magnitude of benefit varies depending on the specific organ and dose metric considered.

### Efficiency of treatment

Delivery efficiency parameters are summarised in Table 2 to facilitate direct comparison across techniques. A clear distinction was observed between C-arm based techniques and helical tomotherapy. VMAT demonstrated a substantial reduction in monitor units compared with IMRT, resulting in shorter beam-on times and improved overall treatment efficiency [16, 17]. In clinical practice, both techniques required only a few minutes per fraction when setup and image guidance were included, with VMAT consistently remaining the faster approach. In contrast, helical exhibited a strong dependence of delivery efficiency on field width. Narrower field widths resulted in prolonged beam-on times, while increasing the field width significantly reduced treatment duration. Despite this improvement, tomotherapy delivery times remained longer than those of C-arm techniques [18, 19]. Additionally, the requirement for MVCT imaging contributed to overall treatment time, further extending the duration compared to CBCT-based workflows. From a clinical perspective, shorter delivery times may reduce intrafraction motion and improve patient comfort, while longer, continuous helical delivery may offer benefits in dose homogeneity and organ-at-risk sparing [20].

**Table 2:** summarises delivery efficiency across IMRT, VMAT, and helical plans. Values are presented as mean ± standard deviation with corresponding ranges. Image-guidance time represents the approximate CBCT or MVCT acquisition time and was included to contextualise the total per-fraction treatment duration

Technique	MU / Beam-on time parameter	Mean ± SD	Image-guidance time	Estimated total treatment time
IMRT	MU	912.0 ± 45.3	CBCT ~ 120 s	~ 5–8 min
VMAT	MU	489.9 ± 52.5	CBCT ~ 120 s	~ 3–5 min
1 cm	Beam-on time (s)	502.7 ± 11.1	MVCT ~ 220 s	~ 12–13 min
2.5 cm	Beam-on time (s)	239.8 ± 10.1	MVCT ~ 220 s	~ 7–9 min
5 cm	Beam-on time (s)	139.8 ± 6.5	MVCT ~ 220 s	~ 6–7 min

### Phantom validation

Measured pacemaker doses on the RANDO phantom surface and at 1 cm depth agreed well with TPS-calculated values across all delivery techniques (Table 3). For IMRT, the mean measured doses were 1.27 Gy (surface) and 0.95 Gy (inside), corresponding to deviations of + 5.83% and + 5.33% from the TPS values (1.20 Gy and 0.90 Gy, respectively). VMAT plans exhibited the largest differences, with measured values of 1.00 Gy (surface) and 0.74 Gy (inside) that deviated by − 9.09% and − 7.25%, consistent with the known TPS underestimation of scattered dose in far-out-of-field regions. Helical TomoTherapy plans demonstrated the best agreement, with deviations below ± 4% for all field widths: − 2.95% (1 cm), − 2.12% (2.5 cm), and − 2.40% (5 cm) for surface readings, and − 3.38%, − 3.00%, and − 3.60% for 1 cm depth, respectively. Overall, the mean measurement–calculation discrepancy across all modalities was within 6% ± 3%, confirming satisfactory dosimetric accuracy of the TPS in the out-of-field dose region. 

**Table 3:** Compares TPS-calculated and OSLD-measured doses at the surface and 1 cm depth across different treatment techniques. Measured values represent the mean of five independent OSLD readings at each position. All doses are reported in Gy. Deviation (%) denotes the percentage difference between TPS-calculated and measured values, calculated using unrounded averages

Plan	TPS Surface (Gy)	TPS Inside (Gy)	Measured Surface (Gy)	Measured Inside (Gy)	Deviation Surface (%)	Deviation Inside (%)
IMRT	1.2	0.9	1.27	0.95	5.83	5.33
VMAT	1.1	0.8	1	0.74	-9.09	-7.25
1 cm	0.95	0.65	0.92	0.63	-2.95	-3.38
2.5 cm	0.85	0.6	0.83	0.58	-2.12	-3.00
5 cm	0.75	0.5	0.73	0.48	-2.40	-3.60

## Discussion

The present study provides a comparative dosimetric evaluation of C-arm based techniques and Helical tomotherapy using three field widths (1 cm, 2.5 cm, and 5 cm) in patients with implanted cardiac pacemakers. All evaluated techniques achieved comparable target coverage. However, smaller tomotherapy field widths, particularly the 1 cm configuration, were associated with lower CIED and thoracic organ doses, albeit with increased treatment delivery time. Because all five plans were generated from the same patient datasets, the PTV–CIED geometry remained identical for each comparison. Therefore, the observed dosimetric differences are more likely attributable to the applied delivery geometry and planning strategy rather than anatomical variation alone.

### Clinical significance and novelty

Earlier studies by Hurkmans et al. [21] and Solan et al. [22] focused primarily on safety thresholds and incidental exposure, while more recent reports [23, 24] evaluated individual platforms in isolation. The present study extends this evidence by demonstrating that helical tomotherapy, particularly with intermediate field widths, can achieve meaningful reductions in CIED dose while maintaining clinically acceptable delivery efficiency. These findings are relevant given the established association between device malfunction and cumulative dose, particularly at cumulative doses exceeding 2 Gy. Although all techniques maintained CIED dose within the low-risk threshold defined by AAPM TG-203 [3] the additional dose reduction observed with tomotherapy provides a clinically relevant safety margin. This may be particularly beneficial in patients with higher-risk devices or prolonged treatment courses, where cumulative scatter dose becomes a concern. Overall, the improved dose control observed with helical delivery supports its role as a valuable option for enhancing procedural safety in pacemaker-bearing patients.

### Comparison with published literature

The present results are consistent with Zhang et al. [25], who reported improved conformity and reduced peripheral dose with tomotherapy, and with Xu et al. [26], who observed improved lung sparing with helical delivery compared with VMAT. In the present cohort, all patients had left-sided pacemakers, representing a clinically challenging ipsilateral geometry. Despite this, helical tomotherapy maintained lower generator doses while preserving target coverage and OAR sparing. These findings are supported by HRS [27] and JASTRO [28], which report low malfunction rates for modern pacemakers and ICDs when cumulative generator dose remains within low-risk ranges. Overall, this study extends prior evidence by linking CIED dose behaviour to tomotherapy field-width variation within a multi-platform comparison. The phantom validation further supported the reliability of the treatment-planning estimates for out-of-field CIED dose. OSLD measurements at the surface and 1 cm depth showed good agreement with TPS-calculated values. The largest discrepancy was observed for VMAT, consistent with TG-158 observations regarding uncertainties in out-of-field scatter dose estimation [29].

### Limitations and future investigation

This study has several limitations. First, its retrospective design and modest sample size, although adequate for within-subject comparison, may not fully represent the anatomical variability encountered in patients with pacemakers. Dosimetric assessment was based on treatment planning data and phantom validation without patient-specific in-vivo dosimetry. Therefore, actual CIED dose may vary due to scatter conditions and tissue heterogeneity. The use of a single tomotherapy unit and C-arm LINAC model may also limit generalizability across different hardware generations. In addition, biological risk modelling was not incorporated. Second, all evaluated pacemakers were positioned outside the primary beam, consistent with TG-203 recommendations. Therefore, the findings apply only to non-overlap geometries where the generator receives predominantly scatter and leakage radiation.

Furthermore, IMRT and VMAT beam arrangements were primarily optimized to avoid direct beam entry through the CIED, while beam exit through the device region was not systematically excluded. Although this reflected a standardized and clinically reproducible planning framework, it may have resulted in conservative overestimation of CIED dose for C-arm LINAC techniques. Consequently, the observed differences between platforms should be interpreted within the context of the applied planning strategy rather than as purely intrinsic technological superiority. More individualized beam-angle and arc-geometry optimization may further reduce CIED dose in selected IMRT and VMAT cases.

Third, all tomotherapy plans used fixed pitch and modulation factor settings across field widths. While this ensured methodological consistency and isolated evaluation of field-width effects, it did not explore the full helical optimization space. Additionally, different dose calculation algorithms were used across platforms as part of routine clinical practice, which may have introduced minor variation in low-dose and out-of-field dose estimation.

Future studies should include multi-institutional datasets, real-time in-vivo dosimetry, Monte Carlo verification, and post-treatment CIED function correlation. Adaptive planning, knowledge-based optimization, and machine-learning models may further improve dose prediction and automated safety flagging near device volumes. Evaluation across newer delivery systems, including Halcyon, Ethos, and Radixact Tomo HD, will also help define broader planning benchmarks for patients with cardiac implantable electronic devices [30–32].

## Conclusion

This study demonstrates that all evaluated techniques achieved comparable target coverage, while differences were observed in CIED dose, organ-at-risk sparing, and delivery efficiency. Helical tomotherapy consistently reduced CIED dose and improved normal tissue sparing, particularly for cardiac and lung structures, with the magnitude of benefit varying across field widths. Smaller field widths provided enhanced dose control near the device, whereas larger field widths improved treatment efficiency. However, these dosimetric advantages must be interpreted in the context of treatment delivery time. Helical tomotherapy was associated with longer beam-on time compared to VMAT, which may increase susceptibility to intrafraction motion in thoracic regions. In contrast, VMAT offered superior delivery efficiency, supporting improved motion management and patient comfort. Overall, the findings highlight that no single technique is universally optimal. Instead, treatment selection should be individualized, balancing dosimetric benefit, delivery efficiency, target geometry, and patient-specific clinical considerations. This study provides quantitative insight into the relationship between field width and CIED dose behaviour, supporting a tailored, clinically driven approach to technique selection in patients with cardiac implantable electronic devices.

## Data Availability

Availability of data and material: All data generated or analysed during this study are included in this article.
